# Windows of Openness to Interaction: Towards Constructive Transdisciplinary Environmental Research and Management

**DOI:** 10.1007/s00267-026-02443-y

**Published:** 2026-04-09

**Authors:** Hanneke J. Nijland, Bernadette F. van Heel, Noelle Aarts

**Affiliations:** 1 Radboud University Nijmegen, Nijmegen, Netherlands; 2 Institute for Science in Society, Radboud University, Nijmegen, Netherlands

**Keywords:** Transdisciplinarity, Openness to interaction, First-person experience, Dysregulation, Window of tolerance

## Abstract

To support constructive interaction in a transdisciplinary environmental research and management project, we developed the theory of *windows of openness to interaction*. Inspired by the window of tolerance concept, this theory links increasing openness to decreasing dysregulation. It introduces two nested windows and identifies a specific zone of constructive interaction in which participants are sufficiently open to collaborate. Our framework is based on a triangulation of in-depth interviews with participants in a transdisciplinary biodiversity project, expert consultations, and literature review. From this, we identified dynamic patterns that shape participants’ experiences and interaction needs, particularly in relation to hypo-arousal and hyperarousal. Building on these insights, we formulated interaction needs and practical approaches that help participants move toward greater openness and collaboration. The framework can be used to recognize and address moments of dysregulation or openness, and to foster meaningful conversations about how participants’ experiences influence constructive interaction in transdisciplinary environmental research and management settings.

## Introduction

In environmental research and management, stakeholders with different views and perspectives have to collaborate and innovate on debated topics. Constructive interaction in such cases is often wrongly taken for granted or assumed. Yet, it is not self-evident and instead requires serious and continuous effort in any multi-stakeholder conversation. We studied ways to foster constructive interaction towards biodiversity restoration in a five-year transdisciplinary socio-ecological research experiment in rural, agricultural contexts in the Netherlands. From 2021 onwards, teams of ecologists, management/administration scientists, and social scientists set out to collaborate with local farmers, nature organizations, citizen initiatives, and policymakers to improve biodiversity in three rural areas (Living Labs). Transdisciplinary research starts from the idea that researchers from different disciplines engage in formal and informal interactions with societal stakeholders with the aim of co-developing experiments in the context of complex problem-solving. For these interactions to be successful, it is crucial that scientists step away from the deficit model of interacting with society, which assumes a knowledge deficit among societal actors that should be filled by scientists. Instead, a dialogical way of interacting should be adopted in which different views, values, experiences, and concerns are exposed with the intention to learn with and from each other (Reincke et al., 2020).

From the start, the initiators knew that constructive interaction would require effort: despite conventional wisdom suggesting that heterogeneous groups are smarter and lead to better results and that actively engaging stakeholders and citizens leads to more support, interactions between stakeholders in environmental research and management often show ambiguity, delays, tensions, and even conflict (Aarts, 2018; La Vos et al., 2019; Roux et al., 2017).

Clearly, constructive interaction through reflecting on individual experiences cannot be taken for granted in collaboration towards biodiversity. Thus, there is a need to draw on insights from, amongst others, psychology, sociology, and communication science to environmental management. The constructive interaction concept touches on the idea of psychological safety, described by Edmondson (1990) as a shared belief that a team is safe for interpersonal risk-taking, allowing members to speak up, admit mistakes, or offer ideas without fear of punishment or humiliation. Constructive interaction also resonates with the concept of interaction-flow, as coined by Van Oortmerssen, Van Woerkum, and Aarts (2015), who focus on interaction patterns that take place in natural talk. We add to this literature by studying (constructive) interaction from the perspective of social and psychological needs of the individual interactants and their influence on windows of openness in interaction.

Generally, approaches to studying – or managing – collaboration in transdisciplinary and other complex group settings often focus on relational dynamics, such as diversity, power, and interdependence (Cuppen, 2012; Stacey, 2007; Turnhout et al., 2020), discursive aspects such as content framing, knowledge construction, and goal setting (Bosschaart et al., 2020; Floor et al., 2018; Pascual et al., 2021), and the influence of complexity, context, and space (Przesdzink et al., 2024; Greenaway et al., 2018; Krabbenborg, 2013; Leeuwis & Aarts, 2011). All these foci are relevant for obvious reasons (and have a place in the current research); however, the impact of individual social and psychological dynamics on such transdisciplinary processes that may also prevent nuanced discussion and collaboration remains underexplored. This paper, therefore, focuses on the role of participants’ experiences in achieving constructive interactions in such transdisciplinary research groups. Building on existing frameworks (see *sensitizing concepts*), we aim to generate insights in constructive interaction in environmental research and management by studying the experiences of individual participants when interacting with other participants in transdisciplinary research settings for biodiversity restoration in the Netherlands. The investigation of preconditions, mechanisms, and circumstances for constructive interactions was an explicit part of the study. With this, we add to existing research on complex multistakeholder interactions, while including psychological safety accepting paradox, and interaction flow (Edmondson, 1990; Bresman & Edmondson, 2022; De Carlo, 2012; Van Oortmerssen et al., 2015).

In the context of science–society interaction, our study zooms in on multistakeholder dialogs as an alternative to the criticized deficit model. The goal of this study is to understand conditions for constructive interactions in transdisciplinary groups working together towards biodiversity. We do so by developing a grounded model that we call windows of openness to interaction. We break down the key aspects and interaction needs involved in openness and dysregulation (i.e., impaired regulation of attention and emotions), distinguishing approaches to regulating individual experience, and using the patterns found to categorize concrete practices that improve constructive outcomes of interactions at the group level.

Our findings highlight the relevance of taking into account that individual openness to interaction co-shapes the quality of the interaction process and its outcomes, nuancing commonly accepted prerequisites such as experiencing safety, respect, and being listened to. The resulting framework provides a tool for (self-)analysis and directions that are applicable in practice to improve facilitation of transdisciplinary projects and move toward more constructive interaction.

## Sensitizing Concepts

The main research question addressed in this paper is: What aids or prevents constructive interaction in transdisciplinary collaboration towards biodiversity? Several starting points and sensitizing concepts (Blumer, 1954) from various research fields provided initial direction toward answering this question.

We see an interaction as a process, consisting of at least one meeting, but typically a series of meetings, in which a group of interactants gathers to achieve individual and, ideally, collective goals. We conceptualize ‘constructive’ as denoting something experienced by interactants as helpful or useful for reaching these goals. We see groups as dynamic wholes, in which the interdependence among members can vary. Like any diverse group, transdisciplinary groups – comprised of scientists and societal actors – form when the integration of differences between interactants is considered essential to reach goals in complex and uncertain contexts (for a similar dynamic and interdependent perspective, see Aarts 2018).

We understand both intrapersonal and interpersonal goals to be dynamic, based on an interplay between past experiences, future projections, and the interpretation of (social and structural) situational information (Aarts & Van Woerkum, 2006). We assume that multiple competing goals of varied relative strength may be active at any given time (Lindenberg & Steg, 2007; Nijland, 2016) and conceptualize that the strength of activated goals increases with emotional charge and identification (Nijland, 2016). We thus see interactions as negotiations comprising two or more interactants who themselves comprise a dynamic dialogical self: a multiplicity of inner goals and thoughts, emotions, and physical sensations that become primed into a certain mindset or identity at a given juncture (Hermans & Hermans-Kanopka, 2010; Nijland, 2016; Slavin, 1996). The frequently made distinction that goals may relate to the content (the topic of conversation), the involved relations (images of ourselves in relation to others, including their different positions), and the interaction process (the course and nature of the communication) (Aarts & Van Woerkum, 2006; Dewulf et al., 2009). formed the starting point for our categorization of aspects of experience.

A related sensitizing concept is ambivalence: the state of having simultaneously activated contradictory goals, thoughts, and feelings, and being unsure which to favor. The degree to which conflicting inner forces are experienced as discomforting, like in music, depends on one’s arousal preference and on whether dissonant instances are followed by their harmonic equivalents (Makowski & Epstein, 2012). Cognitive dissonance theory posits that uncomfortable dissonance usually coincides with the tendency to strategically reduce or avoid it (Festinger, 1964; Janis & Mann, 1977; Serpell, 1996). A less commonly acknowledged, creative way to deal with dissonance is to acknowledge, accept, and even embrace dissonant inner forces from a position of awareness, instead of trying to diminish or dissolve them (De Carlo, 2012; Nijland, 2019). In the short term, the conscious awareness that this state requires might cost a bit of effort, but, in the long term, it may actually save the energy needed to sustain reduction and avoidance strategies.

Similarly, in a group, when interactants have an incongruence of goals or perspectives, there will likely be a tendency to avoid or reduce the discomfort that this may induce in (some members of) the group. Identifying various stages of group development, Gottman et al. (2015), Tuckman (1965), and Lenaerts (personal comment) argue that there is a progressive component to this: with increasing maturity in the group, the ability to accept and embrace difference in connection increases, conflict is sensibly followed by repair, and the need for facilitation decreases. However, when groups have just formed or interactants are constantly changing, facilitation and well-designed interaction methods are usually needed to create the conditions to handle differences constructively.

To create such conditions in transdisciplinary settings and prevent people, goals, and perspectives from being excluded, many authors have provided models, methods, and recommendations (e.g., Aarts, 2015; Brown, 2006; Covey, 1989; Moore, 2014; Pearce & Littlejohn, 1997). We try humbly to add to these by focusing specifically on uncovering mechanisms of individual experiences, while simultaneously viewing the individual and the collective as both interrelated and irreducible parts of any interaction. In doing so, we further build on the window of tolerance (i.e., optimal range of arousal or intensity) concept from psychology (Siegel, 1999) in the specific context of transdisciplinary research for biodiversity restoration.

During our research, we encountered additional theories and concepts – from both literature and experts – that helped us make sense of what we observed. Where relevant, we introduce these in the result section of the paper, in which a theory of individual social and psychological dynamics in multi-stakeholder interactions towards biodiversity is gradually being developed.

## Research Design

We applied an interpretative case-study methodology (Yanow & Schwartz-Shea, 2006) in our research, combining a predominantly phenomenological and hermeneutical analysis of what constitutes constructive interactions in multi-stakeholder interactions. Our approach is phenomenological because it is aimed explicitly at understanding first-person experiences of interactions and hermeneutical because it focuses on the interaction as the unit of analysis and explicitly includes the implications of this phenomenological experience for the quality of the dynamics and the outcome experienced at the group level.

We studied the impact of individual social and psychological dynamics in transdisciplinary research projects that are part of a large research program, ‘Living labs for the restoration of biodiversity in rural areas in the Netherlands’. In this program, Living Labs have been set up in three regions in the Netherlands, where research takes place in which scientists from various disciplines collaborate with stakeholders in the field, mostly farmers. These regions are Ooijpolder-Groesbeek in the eastern Netherlands, the centrally located Alblasserwaard, and the Bollenstreek (Bulb Region) in the western Netherlands. The three living labs reflect a diversity of biodiversity challenges and various durations of transdisciplinary research. Our study coincided with the start of this project, to which we had access through our network.

Our research took place from June 2021 to June 2022 and involved triangulation of insights from (1) 15 semi-structured in-depth conversations of approximately 1½–2 hours each with Living Lab participants from various backgrounds, (2) 15 consultations with communication/facilitation experts selected through snowballing, and (3) literature research to provide sensitizing direction for interpretation and to increase sense-making. Participants in the interviews were selected based on diversity and availability, spread across the three regions. The communication/facilitation experts were chosen for their recognized expertise in multi-stakeholder interaction.

The in-depth conversations were semi-structured, involving questions about respondents’ ambitions and motivations, their perceptions of the interactions that took place in the context of the Living Lab, and their personal lived experiences. These included the perceived success of interactions, the conditions and limiting factors for collaboration, desired outcomes, relationship development, and the interaction process itself. Finally, questions were asked about tensions and ways of dealing with tensions, both by others in the project and internally. A live conversation summary was presented on an online whiteboard during the conversation (visible to, and corrected by, the respondent), and recordings were made as a backup. In all cases, informed consent was obtained to record data and process relevant personal details. The consultations with communication/facilitation experts focused directly on conditions for constructive interactions and were recorded in fieldnotes.

Data were analyzed using an interpretive process called ‘abduction’ (Reichertz 2007; Yanow & Schwartz-Shea 2006): on the basis of continuous interaction and iteration between listening to the reported experiences of people in the field, incorporating expert knowledge, interpreting and reflecting with the help of sensitizing concepts, categorizing and conceptualizing, and revisiting half of the respondents to verify whether findings and our model-in-the-making resonated with their experience and to seek supplementary input. Rather than presenting the results as a thick description (or as unstructured lists of mentioned conditions), while interpreting our data we gradually developed a model in which we distinguished categories of needs, approaches, and practices to better display the perceived patterns. We validated our model in follow-up 1:1 conversations with half of the previously Living Lab participants and communication/facilitation experts.

In the following section, we present the unfolding of a theory (Bowen, 2006) of windows of openness to interaction with the help of quotes that are illustrative for patterns we found through analysis of the in-depth conversations with participants and experts. The result sections are structured as follows: we first introduce the model of windows of openness to interaction. Next, we describe our findings on group dynamics following collaboration from individuals being more or less open to interaction. Next, we describe the mechanism on shifting openness to interactions and finally the needs that have to be met in order to be open to interaction.

## Windows of Openness to Interaction

In psychology, the window of tolerance concept (Siegel, 1999) denotes the zone of arousal in which a person is still able to process mentally and emotionally what is happening without disrupting the functioning of the system (body). This window differs per person: for some people, high degrees of intensity feel comfortable and allow them to think and act effectively, whereas for others even mild degrees of arousal may impair functioning. The window of tolerance can also vary in width, depending on the circumstances: when someone is feeling low in energy for some reason, or overwhelmed by other tasks, they may have a narrower available window of tolerance for a particular task. The basic sensitizing idea here is that everyone has a zone between hypo-arousal (underwhelming) and hyperarousal (overwhelming) in which they are able to endure a certain situation – or interaction – and that there are personal and situational tipping points beyond which they naturally disconnect.

Within the window of tolerance, we argue that these two effects continue and that persons feel increasingly able to interact toward personal optimal arousal levels, i.e., when there is enough arousal to feel active and motivated, yet not so much that stress, agitation, and anxiety overwhelm their ability to interact. From our findings, we propose to add two windows, nested inside the window of tolerance. The first added window we call the window of contribution, where an individual is not just tolerating and attending the interaction, but actively participating in it, and, nested within that, we noticed a window of transformation. Within this window, people are so open to connecting with others and their perspectives that they potentially allow themselves and their ideas to be changed by them; this is associated with a creative state that Van Oortmerssen et al. (2015) dubbed interaction flow:

*“You know, when you are ‘in the zone’: calm, focused, and alert, effortlessly motivated, and building on one another’s words. You just feel totally at ease and energetic at the same time. And you usually come up with great ideas*”.^1^

**Fig. 1:**
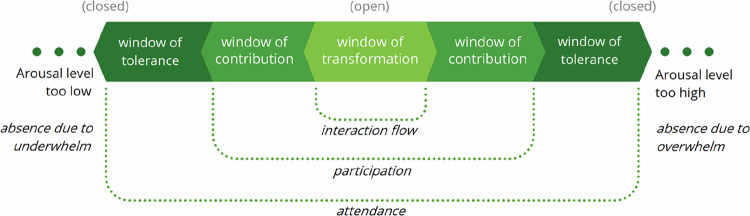
Windows of openness to interaction

We call the resulting model (Fig. 1) the windows of openness to interaction because, toward the center of the spectrum, people report experiencing increasingly more headspace to connect with, listen to, learn from, and attune to the other,^2^ as well as an improved ability for clear, constructive, and authentic expression. Conversely, toward the edges of hypo- or hyperarousal, with a lack of emotions (indifference, boredom, apathy) at one end and overwhelming emotions (anger, anxiousness, or sadness) at the other, people’s systems become increasingly dysregulated, and they experience bodily sensations like lethargy, changes in breathing, or muscles tensing. This dysregulation makes them feel less able to listen deeply and/or express themselves in ways that land with the other – if at all:

*“I find it hard to focus when I’m just not that interested.”] [“I get so revved up, I just black out and can’t find the words. Sometimes it gets so bad that I can’t even communicate that this is going on”*.

Outside the window of tolerance, attending the interaction is very difficult, and one is - usually physically, but certainly emotionally - absent and closed to interacting. At the left end of the spectrum, the interaction and its goal simply have too little priority to care about attending (underwhelming); at the right end (overwhelming), there either is too much else going on or the interaction itself is considered too stressful to attend (for several possible reasons that we will soon elaborate). Though strong emotions can also be a motivation to interact, it makes regulation and constructive interaction more difficult. Additionally, interaction outside this window is not impossible: someone may be considering joining (again), or they know their presence is desired or their absence felt. The dots in the figure indicate a state in which one may still be in some state of involvement: not actually there, but energetically not completely disconnected from the interaction:

*“I know they want me there, but it simply costs me too much energy”*.

In summary, differences between individuals’ overall width and preferred arousal levels occur with differences in energy levels (lots of energy equals wider windows) and arousal preference (more harmony-seeking vs more confrontational); but, for everyone, a certain interaction elicits personal levels of arousal and related openness that can be marked on their specific spectrum.

## Relevance for Constructive Interaction at Group Level

Individual openness to interaction does not necessarily mean that there will be an actual interaction, let alone collaboration or – the reason many partnerships are created – synergy. For that, we argue, there needs to be an overlap in openness to interaction between the people in an envisioned partnership. If the level of openness of each person involved in an interaction is within the window of contribution, collaboration toward a goal usually ensues. Synergy – collaboration in which something (co-)creative emerges, something that is more than the sum of the contributed efforts – seems to occur when interacting individuals are within their windows of transformation: when they are open enough to reach the creative state of interaction flow and potentially allow themselves and their ideas to be transformed during the interaction. This synergetic state of mutual generative listening is generally described as very pleasant, but also as hard to sustain:

*“It’s near magical, but it’s extremely tiring to be there all the time”*.

**Fig. 2:**
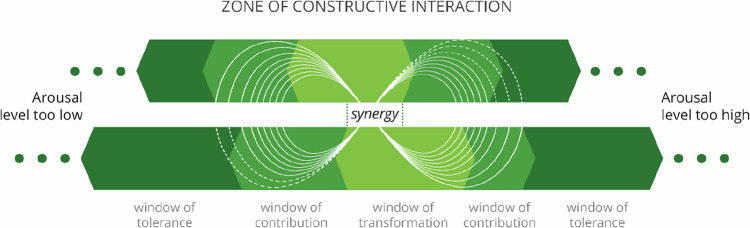
The zone of constructive interaction

Overall, we conceptualize that an interaction is constructive when openness to interaction is oscillating within most partners’ windows of tolerance. The white lemniscates in Fig. 2 depict such a zone of constructive interaction for two partners. In settings with more partners, the picture obviously becomes increasingly complex, but the principle is the same: where participants experience sufficient overlap in windows, collaboration and synergy can occur.

As the dotted white lemniscate lines indicate, temporarily leaving an individual person’s window of collaboration to accommodate another’s can still be part of an overall constructive experience: despite feeling more closed to the interaction and despite requiring some energy, one will usually be able to stay in the momentum of the collaboration. Such brief moments of feeling slightly bored or agitated can usually be tolerated as long as there is movement, and, ideally, recurring moments of experienced synergy. However, when one finds oneself in a more persistent state of dysregulation, trying to sustain collaboration is very difficult – and forcing oneself to stay open is arguably unhealthy.

Following our model, several instances may cause such under- or overarousal and inhibit constructive interaction at the group level. First of all, when one or more partners have a low energy level (e.g., because of lack of sleep, illness, or being overworked), all their windows narrow – leaving less space in which they can be sufficiently open to interaction. Healing and recharging (widening their windows) would be the desired course of action so that they could contribute to interactions more sustainably. For example, one may have so many other (personal or work) activities that feel urgent or important that one experiences limited time/energy for an interaction whose priority one does not (yet) realize. Here, it could be said that the priority of interacting needs to be made clear; but, when one is (almost) absent, a case could also be made for trying to relieve some of one’s other tasks, because an agenda conflict does not necessarily mean that one does not want to be there. There could also be such a mismatch of arousal preferences for openness that interactants’ windows of collaboration hardly overlap - let alone their windows of transformation, meaning that they would have to adapt too much for the interaction to be experienced as constructive at both ends. In that case, they are arguably better off finding teammates with better-matching temperaments.

Typically, however, forces related to the interaction itself cause one or more interaction partners’ arousal levels to move uncomfortably outside the oscillating zone of constructive interaction, resulting in a lack of harmony in the group. In the rest of this paper, we therefore focus more in-depth on these forces by distinguishing mechanisms of shifting openness that emerge in relation to the first-person experience of interactions, as well as on how to better address these in transdisciplinary settings.

## Understanding Mechanisms of Shifting Openness To Interaction

During our research, we found that our respondents showed little difficulty in intuitively indicating the window on the arousal line in which they felt themselves at a certain point in a certain interaction. Moreover, in many cases, they could formulate what would (or did) help them achieve more openness – and whether that was possible at all. We elaborate in detail on these regulating practices and the needs that they address. In general, from left to center in Fig. 2, stimulating practices are needed to conjure motivation and meaning; and from right to center, there is a need for calming practices to enable interactants to experience safety, control, and belonging. However, to be able to do so in a more systematic and practically useful way, we first share some patterns that we found that help to better understand reasons for, and results of, variations in arousal level.

## Aspects of Participants’ Experiences of an Interaction: Content, People, Form, and Each Person’s Own Activities

First of all, a deeper inquiry suggested that individual openness to interaction is more nuanced: respondents could feel open in one way and closed in another. Categorization of our data led us to distinguish four important and interrelated aspects of individual experience, each distinctly contributing to the dynamics of overall openness. In addition, these main aspects may render different degrees of openness at different scales of perception, ranging from macro (project) to micro (meetings or even agenda points). The resulting core aspects of experience, relevant scales, and reasons why they may cause underwhelming in some people and overwhelming in others include:

1. ***Experience of the content of the interaction*** – ranging from the perceived larger issue (usually stated in terms of a project objective – e.g., biodiversity restoration or achieving transdisciplinary collaboration – (macro), to a particular gathering’s perceived topic and goal (micro). In short: How do I relate to what this group wants to do and achieve? This may cause underwhelming because one is indifferent, uninterested, or bored by this content; and overwhelming because one finds it confusing and unclear, too big or complex to deal with, does not deem that the content of the interaction is leading to results fast enough, or considers another topic to have higher perceived urgency or efficacy:
  *“Yes, we need to improve biodiversity, but we don’t need more ecological research, we need to make collective value-based choices and encourage actual change in the field!”*
  Notice the importance of scale: even if one can support the larger issue (macro), a particular meeting’s topic (micro) can create a differing level of arousal.
2. ***Experience of the people involved in the interaction*** – ranging from the general organizational or group culture (macro), to the particular proposed interaction partners and their role, stance, contribution, and behavior (micro). In short: How do I relate to the group of people involved? This may cause underwhelming because working with these people may feel boring, the group or people in it may not feel like the right fit for the goal of the interaction; and overwhelming because the group culture comes across as rigid or intimidating, or one may feel uncomfortable with specific others’ personalities, perspectives, or behaviors, and for example not feel heard, welcomed, or respected by them, or feel intimidated by their role and/or skill.
3. ***Experience of the form of the interaction*** – ranging from the design, planning, and larger infrastructure of the larger project (macro), to the proposed meeting space, facilities, interaction method, and time management (micro). In short: How do I relate to how and where the interactions are organized? This may cause underwhelming because of a lack of affinity with a certain approach, context, or space, or because of finding the process tedious or too slow; and overwhelming because of finding an approach or a work form too challenging, too intense, moving too fast, or finding a setting or space too noisy or otherwise stimulating.
4. ***Experience of one’s own activities for the interaction*** – ranging from a general willingness to contribute to the interaction (macro), to specific (assigned or self-initiated) roles and tasks (micro); these are linked to whether one intrinsically wants to, has the ability to, dares to, or feels obliged to perform the activities and/or perceives them as useful and otherwise rewarding. In short: How do I relate to what I am doing or asked to do for this interaction? This can cause underwhelming because of finding the required or performed activities boring, unrewarding, or not challenging enough; and overwhelming because of feeling pressured to perform them (for example, based on a request from someone with more authority or on whom one is dependent), suffering self-doubt, finding a task too challenging, or requiring too many resources (time/energy/money).

Of course, all categorization is ambiguous to a certain extent: the abovementioned aspects of experience are obviously interrelated, and other (sub-)categories can be made.^3^ However, as these four categories emerged as distinctly important during many interviews, we argue that, for the purpose of understanding where individual openness comes from and for formulating meaningful recommendations for its regulation in groups, these categories provide a practical balance between nuance and simplicity.

## The Dynamic Nature of Participants’ Experiences

Although all aspects and scales (up to the larger context or Zeitgeist) influence individual experience of an interaction and the resulting overall openness to it, we found that at different junctures the relative focus put on them may differ, both between individuals (interpersonal) and within the same person (intrapersonal) – something that we relate to their situational mindset. As stated in the sensitizing concepts, we conceive mindsets to be dynamic: internal and external triggers can activate various inner goals, thoughts, emotions, and physical sensations, based on past experiences, future projections, and the interpretation of social and structural situational information (Aarts & Van Woerkum, 2006; Hermans & Hermans-Kanopka, 2010; Nijland, 2016). This explains why a person can have a strong affinity with biodiversity restoration and be totally open to transdisciplinary interaction with just about anyone, whereas in a particular situation this same person may be triggered by a fellow interactant’s remark or behavior (external cue) that reminds them of a previous negative experience (internal cue), causing them to tense up and experience less openness to interaction. In the latter case, their usual predominantly content-focused mindset now includes self-protection and more focus on the people aspect, demoting content as the core determinant of their openness. This is usually temporary, but, as we examine more deeply in the ensuing section, our findings suggest that, if this situation is not resolved, it may possibly also affect other aspects, for example, their willingness to do certain tasks.

This also further nuances the conceptualization of the zone of constructive interaction: both the sensitizing concepts and our data suggest that the tipping point between experiencing an interaction as, overall, constructive rather than unconstructive does not seem to rely on there being no dysregulation at all, nor on aspects ‘evening each other out’, but on the dysregulation of each aspect not being too extreme and being temporary, i.e., followed by repair and moments of interaction flow. A way to distinguish between these states, according to one of our respondents, is:

*“It feels positive if it overall gives you energy. If it takes it, you have to think and adjust accordingly”*.

A related dimension worth mentioning is the reported dynamic judgment of the perceived topic and goal of specific (micro-level) interactions in relation to the phase of a project: at early stages of group interaction, people are more likely to accept that the sole aim of an interaction is to build relationships, to design ways of organizing the project, or to learn about ideas and perspectives:

*“We need to build trust first, and include farmers and citizens, so the project becomes truly transdisciplinary, and then create shared a vision”*.

Whereas in later stages frustration occurs when the focus is not on taking action to reach tangible results:

*“We’re almost a year in and nothing is happening, everyone is talking past one another, so we’re not getting anywhere. We need to start dividing responsibilities and do something, or the area’s biodiversity will continue to fall”*.

## Interaction Needs and the Mechanism of Dysregulation Frustration

Of all further nuances of mindset and internal cues that can be deemed to explain the mechanisms of participants’ experiences, we found one to be crucial: specific inner goals, which we call interaction needs.

According to our findings, interactants are open enough to listen empathically, express themselves mindfully, and engage in joint action, when oscillating within the zone of constructive interaction. In the most open state – interaction flow –, respondents report the experience of feeling effortlessly motivated; having a clear, creative, and flexible mind; being a useful and valued part of a group of people whom they respect and trust; and feeling a perfect combination of being themselves and being connected to something bigger than themselves. Although the dynamic nature of the experience of interactions makes it virtually impossible to be continuously in interaction flow, the interactions that surfaced from our research seem reminiscent of this state.

We postulate that – with variations due to temperament – the needs listed below are all more or less knowingly present in interacting individuals but are experienced as contributing to dysregulation when they are insufficiently met in a certain situation. In line with our window model, our research moreover indicates that certain unmet interaction needs are linked with hypo-arousal and others with hyperarousal. Although categorization is never perfect and could have been done in multiple ways,^4^ we present interaction needs here in a way that we consider relevant for understanding shifting openness to interaction, as well as for making sense of, and bringing order to, the myriad regulating practices suggested by our respondents (on which we report, using these categories, later in this paper).

The interaction needs to be distilled from our data, connecting with the underwhelming pertains to motivation and meaning are:

- ***The need to have basic affinity*** - feeling basic enthusiasm for the content, valuing fellow interaction partners, enjoying the form, liking one’s own activities.
- ***The need for open exploration*** - feeling curious about being part of new things, engaging in an open-ended inquiry, craving novelty and new experiences, exploring the unknown.
- ***The need for a sense of ownership*** - feeling engaged, finding an issue or topic important enough to commit to it, feeling loyal to a group or leader, invested in and energized by a method, and responsible for one’s own contribution.
- ***The need to have impact*** - making efficient progress, accomplishing goals, (self-)developing and actualizing, experiencing social effectiveness, achieving results, innovating, mastering, influencing, and changing things (in the outside world, in the interaction, or in oneself).
- ***The need to be appreciated*** - being valued, being celebrated, or rewarded for contributing, including being financially compensated for time/energy put into the interaction.
- ***The need for self-transcendence*** - connecting with, and contributing to, something bigger than themselves, doing good, caring for other – human and non-human – beings/future generations/the earth, making a difference, leaving a legacy, finding meaning.

At the other end of the spectrum, linked to overwhelming feelings, we found interaction needs that relate to safety, control, and belonging:

- ***The need for safety*** - feeling physically and psychologically safe in the interaction, treated fairly, being able to trust others and protect oneself, including one’s livelihood.
- ***The need for clarity*** - being aware of the direction of the project, its boundaries, rules, and the division of roles and responsibilities, so as to know what is expected and fair, understanding the language and narratives used.
- ***The need for a sense of autonomy*** - having a sufficient degree of freedom and power: to be oneself, make choices, set personal boundaries, decide what activities to execute, when, and how.
- ***The need to feel capable*** - feeling sufficiently able to perform adequately and in a timely manner, feeling qualified, confident about one’s knowledge/skills – also known as feeling good enough.
- ***The need for dignity*** - ranging from being acknowledged for one’s existence regardless of one’s role and treated with politeness, basic kindness, and humanity (also often called common courtesy), to being taken seriously or even esteemed for one’s qualities, abilities, role, and/or achievements.
- ***The need for belonging*** - being included, feeling accepted and wanted as a trusted member of the group/project, feeling fully connected in the interaction context while also fully being one’s authentic self.
  Partially already reflected in these needs, but worth mentioning separately, the following three core interaction needs were repeatedly seen on both sides of the spectrum:
- ***The need to be oneself*** - being one’s authentic self, with one’s own values, goals, feelings, vulnerabilities, and intersecting identities, being able to show and express these.
- ***The need to be understood*** - feeling seen, heard, and understood in one’s authenticity, being met where one is, and trusted for one’s good intentions.
- ***The need to be connected*** - meeting and interacting with others of one’s own volition, sharing a space with others, regardless of similarities and differences, sharing feelings, relating.

**Fig. 3:**
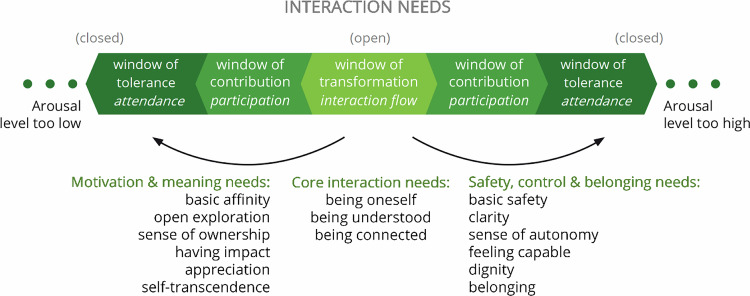
Possibly focal interaction needs

Figure 3 presents a synopsis of interaction needs.

Our research shows that multiple needs, at both ends of the window spectrum, may be unmet simultaneously – and may also be dependent on, or in tension with, one another:

*“I love the project, biodiversity is key. So I want to contribute, achieve something, and feel responsible for it. However, it is still totally unclear to me precisely what our goals are, what everyone’s role is, and what is expected of me, which I find frustrating. We have an amazing team and meet all the time, but don’t get anywhere”*.

Here, clearly, the need for affinity, ownership, and connection is met, but there is a lack of clarity and impact/progress.

In addition, our data suggest that the dysregulation frustration that builds up when a need is unmet for a prolonged time may cause extra resistance as a way of coping, causing more closedness to interaction – possibly even at the other end of the window model:

*“When something bores me and I get confronted with it over and over, I can’t help feeling increasingly irritated”*.

*“When I am constantly stressed out by one person, I soon get stressed by the meetings themselves, and I eventually find myself less and less interested in the topic at hand, even if I used to like it before – almost like it’s an unconscious strategy of my body to prepare for leaving”*.

This mechanism also explains why, although we argue that perceived disrespect at its core is an overwhelming event, underwhelming (or absence because of apparent underwhelming) can become a standard response when the need to feel respected has been structurally unmet:

*“Why would farmers bother coming when they are not taken seriously anyway?”*

*“As soon as I am feeling ignored in a meeting as a woman, I turn blank and sit back. I just am not interested in contributing anymore”*.

In summary, addressing diminished openness to interaction thus calls for the ability to recognize the aspects at play and deal with several, possibly paradoxical, needs at once – and ideally early enough to prevent the formation of unnecessary additional feelings of resistance.

## Discussion and Conclusion

In this paper, we applied an interpretative case-driven methodology on a transdisciplinary research project on biodiversity restoration, and developed a theory that we call windows of openness to interaction. The basic model links states of declining openness to interaction to experiencing increasing hypo- or hyperarousal, divided into three windows, and postulates that there is a zone of constructive interaction in which interactants’ openness is sufficient for differences to be embraced instead of avoided, and (moments of) synergy can occur.

This study contributes a framework with which to foster constructive multistakeholder interactions in transdisciplinary research projects towards biodiversity restoration. At a minimum, our framework can be used to engage in meaningful conversations about the mechanisms of participants’ experiences and how the phenomenon of fluctuating openness to interaction co-shapes the quality of an interaction process and its outcomes. Although relevant in any diverse group, we argue that this is especially important in transdisciplinary settings, where values, priorities, and perspectives differ fundamentally, and (inter)dependencies, power differences, and exclusion are common – often combined with an implicit or even explicit cultural expectation for interactants always to be open to interaction in the name of professionalism. Therefore, especially in transdisciplinary research, a better understanding of these social dynamics in interaction is crucial for co-learning (Roux et al., 2017). More generally, the framework may be of use in conversations with and among non-academic academic actors working in environmental management as well as other fields, stressing the need to further co-develop the framework to fit their practice as well.

From our research, we argue that it is advisable to treat social dynamics and states of being closed to interaction not as a personal failure (or merit), but as resulting from the interaction itself (see also Elias,^1970^). Moreover, these interactions are continuously influenced by external and internal circumstances and developments. Consequently, the states of being open or closed to interaction alternate and are dynamic. Our research shows that, to some extent, this can be regulated by making adjustments in the interactions, an insight that certainly deserves more attention in transdisciplinary projects and other multi-stakeholder collaboration processes.

Our research has a few limitations. It is an exploratory, qualitative study, which entails that the theoretical insights obtained need to be further substantiated and may be further deepened. Further empirical research as well as practical experience is needed, also beyond the Netherlands, to verify our framework and its applications and generate more insight into how interaction processes evolve – both between interactants, inside their heads, and the wider environment. Future research should further develop how interactions can be regulated toward openness among the interactants. We recommend, for instance, looking for additional explanatory mechanisms at the group, organizational, and societal level, because although these are influenced by the smaller directional parts of which they are composed, they also form entities, or processes, with their own dynamics. Moreover, we recommend distinguishing patterns in openness to interaction from mere situational states and investigating the influence of (group) culture on individual participants’ experiences, and the other way around. Additionally, as our research suggests that regulating interactants toward openness does not eliminate conflict, but aids embracing differences within the interaction as fuel rather than obstacles, we suggest that the framework can help researchers consolidate the notion of constructive conflict.

Embracing differences and different perspectives rather than eliminating them is crucial in environmental management for creating engagement, for connecting to a changing environment, for creativity, and for realistic decision making (Van Woerkum & Aarts, 2002). In practice, this could imply collaborative framing of research questions as well as increased attention on embodied interpersonal skills, which are not yet taught structurally in academia but are required for collaborations based on trust, care, and reciprocity (Milberg Muñiz et al., 2024). From our study, we conclude that being able to capitalize on the value of this diversity in environmental management and in transdisciplinary research requires the improvement of stakeholders’ agency and meeting interaction needs for windows of transformation and constructive interaction. In this way, interactions on the ground will help to address, rather than hinder, complex societal changes such as restoring biodiversity.

## Data Availability

No datasets were generated or analysed during the current study.

## Footnotes

1 Bracketed italicized quotes indicate comments respondents made during data collection. Quotes were translated from Dutch into English by the authors.

2 See Scharmer (2008), who discerns between the increasingly attuned downloading, factual, empathic, and generative listening.

3 E.g., further dividing ‘content’ into ‘topics’ and ‘objectives’, ‘form’ into ‘methods’ and ‘infrastructure’ (as the format of an interaction arguably differs from the space and place in which it takes place), or merging ‘one’s own activities’ with ‘people involved in the interaction’ into a larger category of ‘relationships’.

4 E.g., we could have ‘sliced the pie’ in different ways, leading to other (sub-)categories; and we could have made a link that we noticed between needs and the windows of tolerance, contribution, and transformation clearer than we have done by rating them in order of wider to narrower windows.
